# The Naturalist

**Published:** 1845-12

**Authors:** 


### THE NATURALIST.

Such is the title of a monthly publication projected by the Faculty of Franklin College, near Nashville, in Tennessee, which is to be devoted to geology, mineralogy, zoology, entomology, botany, and agricultural chemistry. President Fanning has charge of the department of horticulture and agriculture; the department of natural history is confided to Professor I. N. Loomis. We can answer for the devotion of these gentlemen to the branches of science respectively assigned to them. Mr. Fanning is an ardent lover of the country, and a zealous, persevering, and enlightened friend of agriculture. Mr. Loomis is engaged in the cultivation of geology and its kindred sciences with a zeal which promises much to the natural history of the West. We heartily wish them success in this enterprise, as well as in their laudable effort to blend learning with the cultivation of the soil. Physicians are interested in whatever relates to the science of living beings, and compose a class of readers who would find amusement and instruction in the pages of the Naturalist, Y.

